# Dark Triad and relational aggression: the mediating role of relative deprivation and hostile attribution bias

**DOI:** 10.3389/fpsyg.2024.1487970

**Published:** 2024-11-29

**Authors:** Yongzhi Jiang, Lifang Tong, Wenjiao Cao, Huizhe Wang

**Affiliations:** ^1^Inner Mongolia Minzu University, School of Educational Science, Tongliao, China; ^2^Inner Mongolia Autonomous Region Student Bullying Prevention and Control Research Center, Tongliao, China; ^3^Tongliao Vocational College, Tongliao, China

**Keywords:** Dark Triad, Machiavellianism, psychopathy, narcissism, relational aggression, relative deprivation, hostile attribution bias

## Abstract

**Introduction:**

Relational aggression, as a distinct form of aggressive behavior in social relationships, is associated with various physiological and psychological disorders. Although previous research has provided theoretical support for the connection between the Dark Triad (Machiavellianism, psychopathy, and narcissism) and relational aggression, the mediating factors between the two still require in-depth exploration. This study employed a cross-sectional research method to examine the mediating roles of relative deprivation and hostile attribution bias between the Dark Triad and relational aggression.

**Method:**

This research employed the Dark Triad scale, the relational aggression scale, the relative deprivation scale, and the hostile attribution bias scale to conduct a self-reported questionnaire survey involving 1,968 students from two universities in China.

**Results:**

The Dark Triad traits significantly predicted relational aggression. The mediating role of relative deprivation was significant in the relationship between these traits and relational aggression. Hostile attribution bias mediated the relationship between Machiavellianism, psychopathy, and relational aggression, but not between narcissism and relational aggression. Additionally, the serial mediating role of relative deprivation and hostile attribution bias was significant between the Dark Triad traits and relational aggression.

**Discussion:**

This study not only verified the relationship between the Dark Triad and relational aggression but also identified mediating factors providing new useful information for effectively explaining and intervening in malignant interpersonal relationships.

## Introduction

Relational aggression is a distinct form of aggressive behavior aimed at a person’s social relationships. Experiencing this form of aggression is associated with the development of various physiological and psychological symptoms, including sleep disturbance, alcohol abuse, stress, depression, anxiety, and anger (Dahlen et al., 2013; Deason et al., 2019; Osgood et al., 2021). It is defined as the deliberate use of interpersonal manipulation to undermine and threaten the social status, reputation, or relationships of others, such as spreading rumors, peer threats, intentional neglect, and group exclusion (Crick, 1996; Werner and Crick, 1999; Archer and Coyne, 2005). In the past two decades, the connection between relational aggression and traits of antisocial personality has been widely studied by researchers (Tackett et al., 2013), with the most typical personality factors being the Dark Triad (Giammarco and Vernon, 2014; Jonason and Kroll, 2015). According to the general aggression model, the interaction of individual and situational factors activates the individual’s internal state (cognition, emotion, arousal, and their interplay), which in turn triggers behavioral outcomes (Anderson and Bushman, 2002). Based on this, the link between the Dark Triad as an individual factor and relational aggression may be influenced by situational factors and internal states. However, most of the current research on the Dark Triad and relational aggression has focused separately on situational factors and internal states, such as the pursuit of social status, self-disclosure on social media, hostile attribution bias, and moral disengagement (Bell et al., 2017; Abell and Brewer, 2014; Law and Falkenbach, 2018; Erzi, 2020), whereas few studies explore the complex links between situational factors and internal states in depth. Therefore, this research, by combining situational factors and internal states, aims to delve into the relationship between the Dark Triad and relational aggression, as well as the mediating factors between them. This will not only enrich and expand the research on the Dark Triad and relational aggression but also hold significant theoretical value and practical significance for understanding and intervening in malicious interpersonal relationships.

### Dark Triad and relational aggression

Dark Triad personality traits, namely Machiavellianism, psychopathy and narcissism, share a tendency toward aggressiveness and emotional coldness (Paulhus and Williams, 2002). Specifically, Machiavellianism is characterized by a tendency to manipulate or deceive others for personal gain (Christie and Geis, 1970); psychopathy reflects a cold and impulsive nature in interpersonal relationships and takes pleasure in harming others (Rauthmann, 2012; Erzi, 2020); narcissism often manifests as a sense of superiority, entitlement and vanity (Campbell and Foster, 2010; Morf and Rhodewalt, 2001). Research indicates that females with high Machiavellianism tend to exhibit dishonest self-disclosure and relational aggression toward close friends on social media (Abell and Brewer, 2014); concurrently, due to their inherently unemotional traits, individuals with psychopathic tendencies often employ moral disengagement to carry out relational aggression, thereby reinforcing beliefs in schadenfreude (Erzi, 2020); additionally, studies on narcissism have found that narcissists often use their peer networks as a means to attain social dominance (Fanti and Henrich, 2015), and when they perceive threats to their self-image or popularity within peer relationships, they may respond with aggressive behaviors (Onishi et al., 2012). In a study involving children and adolescents, the unique link between narcissism and relational aggression has been confirmed, suggesting that relational aggression may be a common tactic for narcissists to counter others, gain, or maintain social status (Bell et al., 2017). Based on the above analysis, this study proposes the hypotheses 1: Machiavellianism, psychopathy, and narcissism positively predict relational aggression.

### The mediating role of relative deprivation

Most contemporary theories and research support the notion that human behavior is the result of the interaction between personality and social context (Zagenczyk et al., 2017). In social relationships, when individuals perceive themselves as treated unfairly or unjustly, they may feel that others have deprived them of their rights or benefits that should have been rightfully theirs; this subjective perception is termed relative deprivation in the field of social psychology (Stouffer et al., 1949; Bernstein and Crosby, 1980). According to the theory of relative deprivation, individuals in an unfavorable social position are likely to compare themselves with others. If they feel deprived in comparison, they may respond with negative emotions like anger and resentment, which can lead to deviant behaviors (such as aggression, violence, theft, etc.) or withdrawal behaviors (such as alcoholism, smoking, substance abuse, gambling, etc.) (Smith et al., 2012). Pavlovic and Franc (2021) found that individuals with the Dark Triad traits may experience a heightened sense of deprivation when faced with unfair treatment or infringement of interests, potentially escalating to extreme behaviors. Specifically, Machiavellianism’s self-interest and manipulative traits can trigger feelings of deprivation and extreme behavior when benefits are lost (Pavlovic and Storm, 2020). Similarly, the psychopathy and narcissistic superiority driven by the pursuit of self-satisfaction can evoke feelings of deprivation and aggressive intentions when their ideal expectations are thwarted (Walsh et al., 1987; Zeigler-Hill and Dehaghi, 2023). Despite numerous studies indicating that relative deprivation is a key predictor of aggressive behavior (Zhou, 2023; Zhang and Zhang, 2023; Peng et al., 2023), no research to date has comprehensively examined the link between relative deprivation and relational aggression. Based on this, this study proposes the hypotheses 2: Relative deprivation positively predicts relational aggression and plays a mediating role between Machiavellianism, psychopathy, narcissism, and relational aggression.

### The mediating role of hostile attribution bias

The implicit personality theory suggests that the beliefs generated by personality traits shape people’s interpretations and encoding of social events, determining how individuals uniquely perceive situations and subsequently respond to different stimuli (Schneider, 1973). According to this theory, the formation and development of relational aggression may be linked not only to the Dark Triad but also to the malicious beliefs associated with the Dark Triad. Hostile attribution bias is defined as the cognitive tendency to interpret others’ behaviors and words as hostile when social contextual cues are ambiguous, and it is a key factor in the etiology of problematic behaviors (Milich and Dodge, 1984). Social information processing (SIP) theory posits that social cues are interpreted according to individual biases and beliefs. Ambiguous cues may lead to complex information processing requiring multiple explanations, whereas hostile attribution bias arises from cognitive deficits in processing (Dodge and Crick, 1990). Some researchers suggest that hostile attribution bias may be more pronounced in psychopathic populations, as their emotional deficits may hinder the perception of non-dominant social cues and the distinction between ambiguous and hostile situations (Maccoon and Newman, 2006). Narcissistic individuals’ aggressive behavior may also be tied to hostile attribution bias. The threatened egotism model suggests that narcissists are prone to negative reactions to challenges to their authority, exhibiting increased hostility and aggression in social settings (Bushman and Baumeister, 1998). Although the link between Machiavellianism and hostile attribution bias is not yet fully established, the self-interest and distrust characteristic of Machiavellianism are likely important contributors to such bias. Existing research confirms that hostile attribution bias is a significant cognitive mechanism in relational aggression (Godleski and Ostrov, 2010). For example, de la Osa et al. (2018) found that children with oppositional defiant disorder and high levels of hostile attribution bias were more likely to engage in relational aggression; similarly, Bailey and Ostrov (2008) identified a unique association between relational aggression in emerging adults and hostile attribution bias in conflict provocation. Therefore, it is likely that the Dark Triad exhibits a stable hostile attribution bias toward the outside world, which may be related to relational aggression. Based on the above analysis, this study proposes the hypotheses 3: Hostile attribution bias mediates the relationship between Machiavellianism, psychopathy, narcissism, and relational aggression.

### The relationship between relative deprivation and hostile attribution bias

Furthermore, some scholars suggest that whereas relative deprivation is a risk factor for aggressive behavior, the psychological processes underlying aggressive behavior related to deprivation require further investigation (Greitemeyer and Sagioglou, 2019). The relative deprivation perspective posits that adverse situations or experiences of deprivation can trigger aggressive behavior through emotions like anger and resentment (Smith et al., 2012). Therefore, relative deprivation may be a distal determinant, whereas hostile emotions are proximal determinants of aggressive behavior. A recent study supports this view, indicating that relative deprivation predicts hostile attribution bias, which mediates the relationship between relative deprivation and aggressive behavior (Xu, 2023). Based on this, this study proposes the hypotheses 4: Relative deprivation positively predicts hostile attribution bias, and relative deprivation and hostile attribution bias play a serial mediating role in the relationship between Machiavellianism, psychopathy, narcissism, and relational aggression.

## Methods

### Participants and procedure

This study used a convenience sampling method to conduct a questionnaire survey of first to fourth-year students at two full-time universities in Inner Mongolia, China. To ensure the validity of the questionnaire and filter out those that were not answered seriously, this study included an attention check question (to test the authenticity of the questionnaire, please select “somewhat agree”). In addition, this study also excluded a portion of questionnaires with obviously unreasonable options. Ultimately, 1,968 valid questionnaires were selected from 2,105, resulting in a valid response rate of 93.49%. Among them, there were 745 males (37.86%) and 1,223 females (62.14%); the age range of the participants was 18–25 years, with an average age of 20.70 years and a standard deviation of 1.460 years.

All procedures in this study were reviewed and approved by the ethics committee of the author’s university. The research was conducted with the informed consent of the participating teachers and school principals. A graduate student specializing in mental health education, with extensive experience in psychological testing, acted as the primary administrator, supported by two fellow students. Before the testing, the research and testing content were explained in detail to the class counselors, who received uniform training and guidance. During break time, the class counselors provided a brief overview of the study to the students, after which the teachers distributed the questionnaire link. Students voluntarily accessed the questionnaire website by clicking the link on their phones and could opt out at any time during the process. Given the reliance on self-reported data, participants might have been influenced by social desirability biases, potentially leading to more socially acceptable responses. To mitigate this, the questionnaire was designed to be anonymous, and the researchers assured participants that their information would be kept confidential.

### Measures

#### Dark Triad

The study employed the Short-Dark Triad (SD3) revised by Chinese scholar Geng et al. (2015). This scale consists of 27 items, including three subscales, each containing nine items that correspond to the three personality traits of the Dark Triad: Machiavellianism (1. It is unwise to reveal your secrets...), Psychopathy (1. I enjoy challenging losers...), and Narcissism (1. I like to be the center of attention...). A 5-point Likert scale was used (1 = strongly disagree, 5 = strongly agree), with higher total scores indicating higher levels of personality traits in each dimension. The scale has demonstrated good reliability and validity, making it appropriate for use with college students (Shi et al., 2022). In this study, the Cronbach’s alpha coefficients for Machiavellianism, Psychopathy, and Narcissism were 0.866, 0.750, and 0.629, respectively.

#### Relational aggression

The study utilized the Relationship Aggression Behavior Scale revised by Chinese scholar Liang (2006). The scale consists of nine items (1) when someone makes me angry, I will ignore him/her for a short time. (2) When someone makes me very angry, I will angrily tell my friends about that person’s shortcomings. (3) If someone is always opposing me, I will tell him/her that if it continues, I will expose his/her privacy. (4) When I have a conflict with a same-sex friend, I will be little him/her in front of others..., using a 5-point Likert scale (1 = strongly disagree, 5 = strongly agree). The higher the total score after summing the scores, the higher the individual’s level of relationship aggression behavior. This scale has demonstrated good reliability and validity in previous research with college student samples (Xia et al., 2017). In this study, the Cronbach’s alpha coefficient for the scale was 0.720.

#### Relative deprivation

The study employed the Relative Deprivation Scale developed by Chinese scholar Ma (2012). This questionnaire measures individuals’ subjective perceptions of deprivation arising from comparisons with relevant reference groups. It consists of four items: (1) My life should be better than it is now compared to the efforts and contributions I have made. (2) I always feel that others possess things that should belong to me. (3) Compared to those around me, I am at a disadvantage in various aspects of life and work. (4) Most wealthy people in society have made their fortunes through unscrupulous means. A 6-point Likert scale was used (1 = strongly disagree, 6 = strongly agree), with higher total scores indicating a higher level of perceived relative deprivation. The scale has been found to have good reliability and validity for college students (Hu and Xiong, 2024). In this study, the Cronbach’s alpha coefficient for this questionnaire was 0.716.

#### Hostility attribution bias

The study utilized the revised Chinese version of the WSAP-Hostility Scale, developed by Chinese scholar Quan and Xia (2019). This scale includes two dimensions: benevolent attribution bias and hostile attribution bias, with this study focusing on the hostile attribution bias section. It consists of 16 items (1) someone slams the door in front of you, and you perceive this behavior as insulting. (2) A friend makes a joke at your expense, and you consider this behavior disrespectful. (3) Someone frowns at you, and you view this behavior as hostile. 4. A friend declines your dinner invitation, and you think this behavior is rude..., using a 6-point Likert scale (1 = strongly disagree, 6 = strongly agree). The total score is calculated by summing the scores, with a higher total indicating a higher level of hostile attribution bias. The scale has demonstrated good reliability and validity for college students (Li and Xia, 2021). In this study, the Cronbach’s alpha coefficient for the scale was 0.908.

### Data analytic procedures

This study employed SPSS 24.0 and Mplus 8.0 for data processing and mediation effect testing. The specific analysis approach is as follows: (1) Descriptive statistics and correlation analysis for each variable’s data were conducted using SPSS 24.0; (2) Mplus 8.0 was used to test: the mediating role of relative deprivation in the relationships between Machiavellianism, psychopathy, narcissism, and relational aggression; the mediating role of hostile attribution bias in the relationships between Machiavellianism, psychopathy, narcissism, and relational aggression; and the serial mediating role of relative deprivation and hostile attribution bias in the relationships between Machiavellianism, psychopathy, narcissism, and relational aggression. Considering the parameter testing phase, if the data are not normally distributed or if there is heteroscedasticity, it may lead to an increase in Type I and Type II errors. Therefore, this study adopted the bias-corrected non-parametric percentile Bootstrap method to test the significance of regression coefficients, as recommended by Erceg-Hurn and Mirosevich (2008). Specifically, 5,000 Bootstrap samples were drawn to determine the standard error of parameter estimates and the 95% Bootstrap confidence interval. If this interval does not include 0, it indicates that the statistical result is significant.

## Results

### Common method bias test and multicollinearity test

Since all data in this study were collected using self-report methods, the potential for common method bias exists (Zhou and Long, 2004). To address this, we conducted Harman’s single-factor analysis. The unrotated factor analysis revealed a total of 10 factors with eigenvalues greater than 1, and the first factor explained only 22.35% of the variance, which is less than 40% threshold, suggesting that common method bias is not a significant concern in this study’s data. We then evaluated the multicollinearity among predictor variables by examining the variance inflation factor (VIF). The multicollinearity test results indicated that the tolerance values for all predictor variables ranged from 0.564 to 0.672 (≤0.1 indicates the presence of multicollinearity), and the VIF values ranged from 1.488 to 1.772 (≥10 indicates the presence of multicollinearity), confirming that multicollinearity is not an issue among the predictor variables.

### Descriptive statistics and correlation

Table 1 presents the means, standard deviations, and correlations of various variables. The correlation analysis reveals significant positive relationships between Machiavellianism, psychopathy, and narcissism, and each of these traits is associated with relational aggression, relative deprivation, and hostile attribution bias. Additionally, relative deprivation and hostile attribution bias are significantly positively correlated with each other, as well as with relational aggression.

**Table 1:** Mean, standard deviation, and correlation of each variable (*N* = 1968).

Variables	*M*	SD	1	2	3	4	5	6	7	8
1.Gender	—	—	—							
2.Age	20.70	1.460	—	—						
3.Machiavellianism	3.097	0.769	0.019	−0.029	1					
4.Psychopathy	2.487	0.646	−0.061^**^	−0.051^*^	0.521^**^	1				
5.Narcissism	2.979	0.529	0.019	0.011	0.488^**^	0.495^**^	1			
6.Relative deprivation	2.878	0.878	−0.040	−0.029	0.430^**^	0.538^**^	0.420^**^	1		
7.Hostile attribution bias	3.122	0.873	0.086^**^	−0.013	0.507^**^	0.431^**^	0.353^**^	0.436^**^	1	
8.Relational aggression	2.670	0.538	0.017	−0.011	0.398^**^	0.442^**^	0.362^**^	0.417^**^	0.391^**^	1

^*^p < 0.05; ^**^p < 0.01; ^***^p < 0.001.

### Mediating role of relative deprivation and hostility attribution bias

**Figure 1:**
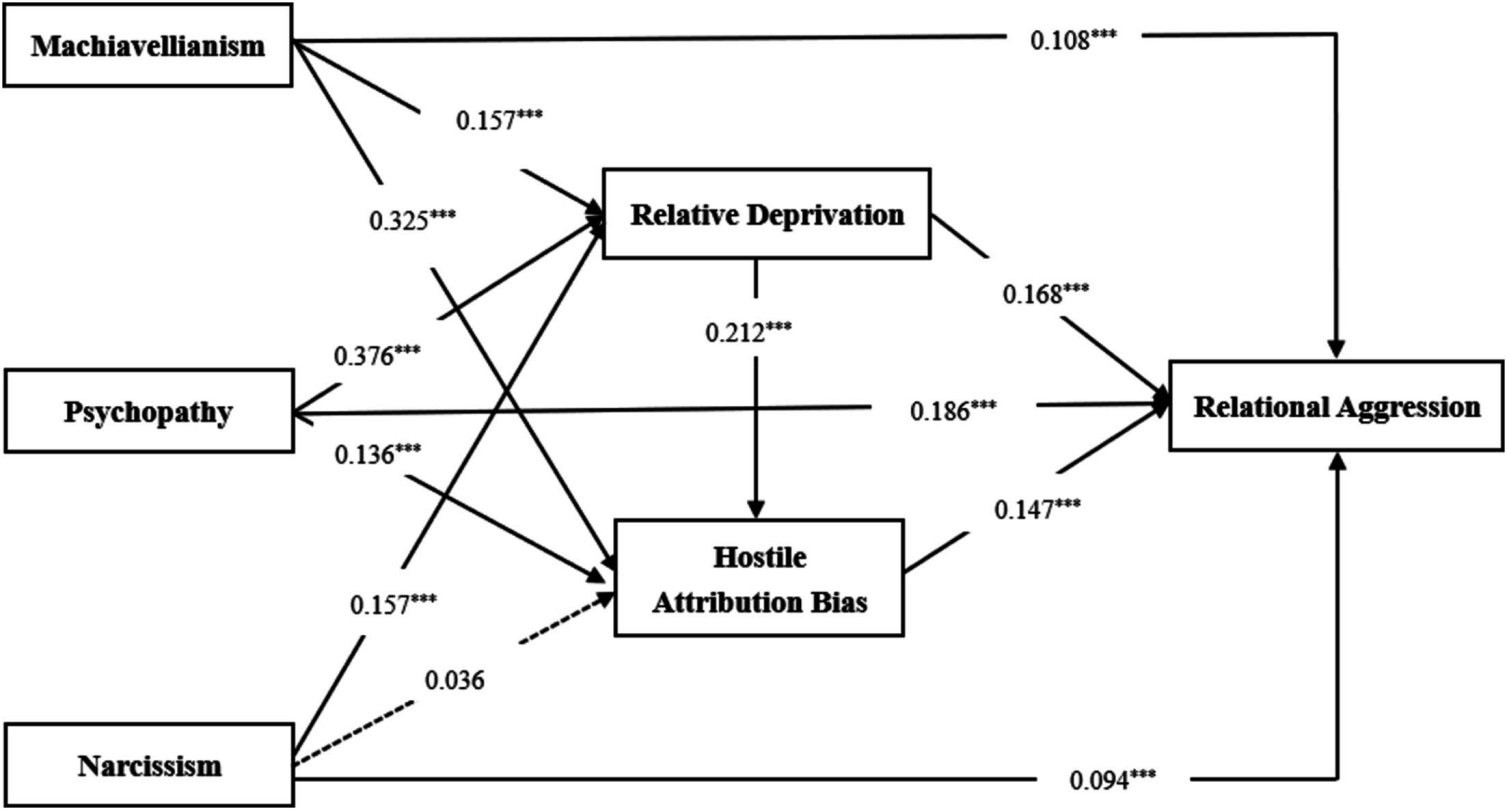
Effect tests and path analysis among various variables.

The correlation analysis results meet the necessary statistical criteria for further testing the mediating effects of relative deprivation and hostile attribution bias (Wen and Ye, 2014). Therefore, we employed the Bootstrap method with 5,000 replicates to validate the mediating effects. Controlling for gender and age, we set Machiavellianism, psychopathy, and narcissism as independent variables, relational aggression as the dependent variable, and relative deprivation and hostile attribution bias as mediating variables. The mediating effect test results are depicted in Figure 1. The findings are as follows: Machiavellianism significantly predicted relational aggression (*β* = 0.108, *p* < 0.001), relative deprivation (*β* = 0.157, *p* < 0.001), and hostile attribution bias (*β* = 0.325, *p* < 0. 001); psychopathy significantly predicted relational aggression (*β* = 0.186, *p* < 0.001), relative deprivation (*β* = 0.376, *p* < 0.001), and hostile attribution bias (*β* = 0.136, *p* < 0.001); narcissism significantly predicted relational aggression (*β* = 0.094, p < 0.001) and relative deprivation (*β* = 0.157, *p* < 0.001) but did not significantly predict hostile attribution bias (*β* = 0.036, *p* > 0.05); relative deprivation significantly predicted relational aggression (*β* = 0.168, *p* < 0.001) and hostile attribution bias (*β* = 0.212, *p* < 0.001); hostile attribution bias significantly predicted relational aggression (*β* = 0.147, *p* < 0.001).

Based on the effect test and path analysis, we conducted a mediation effect test using the bias-corrected nonparametric percentile Bootstrap method. The results, presented in Table 2, indicate the following mediation effects through eight specific paths: Machiavellianism → relative deprivation → relational aggression (indirect effect 1), effect size 0.018, accounting for 13.74% of the total effect; psychopathy → relative deprivation → relational aggression (indirect effect 2), effect size 0.053, accounting for 22.55% of the total effect; narcissism → relative deprivation → relational aggression (indirect effect 3), effect size 0.027, accounting for 20.30% of the total effect; Machiavellianism → hostile attribution bias → relational aggression (indirect effect 4), effect size 0.034, accounting for 25.95% of the total effect; psychopathy → hostile attribution bias → relational aggression (indirect effect 5), effect size 0.017, accounting for 7.24% of the total effect; Machiavellianism → relative deprivation → hostile attribution bias → relational aggression (indirect effect 6), effect size 0.003, accounting for 2.29% of the total effect; psychopathy → relative deprivation → hostile attribution bias → relational aggression (indirect effect 7), effect size 0.010, accounting for 4.25% of the total effect; narcissism → relative deprivation → hostile attribution bias → relational aggression (indirect effect 8), effect size 0.005, accounting for 3.76% of the total effect. Specifically, relative deprivation serves as a significant independent mediator between Machiavellianism, psychopathy, and relational aggression, as well as between narcissism and relational aggression; hostile attribution bias is a significant independent mediator between Machiavellianism and relational aggression, and between psychopathy and relational aggression, but not between narcissism and relational aggression; relative deprivation and hostile attribution bias together play a significant serial mediating role between Machiavellianism and relational aggression, psychopathy and relational aggression, and narcissism and relational aggression.

**Table 2:** Mediating effects of relative deprivation and hostility attribution bias.

	Effect path	Effect value	95%CI	Relative percentage
Sole-mediator effect	X1 → M1 → Y	0.018	[0.013, 0.026]	13.74%
	X2 → M1 → Y	0.053	[0.039, 0.068]	22.55%
	X3 → M1 → Y	0.027	[0.018, 0.038]	20.30%
	X1 → M2 → Y	0.034	[0.023, 0.045]	25.95%
	X2 → M2 → Y	0.017	[0.010, 0.025]	7.24%
	X3 → M2 → Y	0.005	[−0.001, 0.013]	3.76%
Serial-mediated effect	X1 → M1 → M2 → Y	0.003	[0.002, 0.005]	2.29%
	X2 → M1 → M2 → Y	0.010	[0.006, 0.014]	4.25%
	X3 → M1 → M2 → Y	0.005	[0.003, 0.008]	3.76%
Direct effect	X1 → Y	0.076	[0.044, 0.107]	58.02%
	X2 → Y	0.155	[0.117, 0.193]	65.96%
	X3 → Y	0.096	[0.052, 0.138]	72.18%

X1, Machiavellianism; X2, psychopathy; X3, narcissism; M1, relative deprivation, M2, hostile attribution bias, and Y, relational aggression.

## Discussion

Based on the above research findings, this study confirms hypothesis H1: Machiavellianism, psychopathy, and narcissism significantly predict relational aggression. The results suggest that individuals with these traits are more likely to engage in relational aggression. Specifically, Machiavellians, because of their low emotional awareness, may be less influenced by the verbal behaviors of others (Zhao et al., 2023). They are inclined to prioritize self-interest and may perceive relational aggression as a strategy to achieve their objectives. Nevertheless, the link between Machiavellianism and relational aggression is not straightforward, particularly when considering peer relationships and individual differences. For example, Peeters et al. (2010) discovered that women with high levels of Machiavellianism who engage in relational aggression are associated with popularity, social intelligence, and high social exclusion. Surprisingly, men with low levels of Machiavellianism exhibit similar patterns. Thus, the relationship between Machiavellianism and relational aggression may need further exploration within a wider range of contexts.

Simultaneously, this study suggests that psychopathy may be a stronger predictor of relational aggression than Machiavellianism. For example, Warren and Clarbour (2009) found that the cold-heartedness and antisocial impulsivity associated with psychopathy are key triggers of relational aggression. Similarly, Czar et al. (2011) indicated that psychopathic traits maintain a strong predictive effect on relational aggression even after controlling for physical aggression. This suggests a potentially stronger association between psychopathy and relational aggression.

Furthermore, this study confirms the predictive role of narcissism in relational aggression, aligning with previous findings (Baughman et al., 2012). The threatened egoism model posits that individuals have an inherent need for recognition and affirmation from others to validate their self-worth, perceiving anything that threatens it as a potential provocation (Bushman and Baumeister, 1998). Consequently, narcissists may be strongly affected by negative social feedback, prompting them to resort to relational aggression to preserve their social standing. Some scholars argue that narcissism, a multifaceted personality trait, can vary in expression across its subtypes (Gore and Widiger, 2016). For instance, studies have shown that grandiose narcissism, marked by high self-esteem and a sense of entitlement, may act as a buffer against relational aggression, whereas vulnerable narcissism, characterized by hypersensitivity and low self-esteem, may exacerbate it (Knight et al., 2018). Nevertheless, research on the relationship between narcissism subtypes and relational aggression is limited. Future research could benefit from a deeper investigation into this matter.

From a subjective perception perspective, this study reveals that relative deprivation positively predicts relational aggression and serves as a significant mediator between Machiavellianism, psychopathy, narcissism, and relational aggression, thus supporting research hypothesis H2. The findings suggest that individuals high in Machiavellianism, psychopathy, and narcissism are more likely to feel relatively deprived and to engage in relational aggression. Firstly, Machiavellianism is known for its cynicism (the belief in the weakness and untrustworthiness of others) and the pursuit of practical benefits. Individuals with this trait typically manipulate situations to maximize their interests, finding satisfaction in this process. Therefore, when faced with obstacles to their goals in social relationships, they may feel a strong sense of relative deprivation and turn to relational aggression as a coping strategy. Secondly, psychopaths’ relational aggression is tied to their sense of relative deprivation. For example, researchers such as Dawel et al. (2012) conducted a meta-analysis and found that individuals with high levels of psychopathy are more likely to pursue self-gratification with a disregard for others. Thus, when these individuals encounter obstacles to fulfilling their self-gratification goals, external impediments not only cause them to experience feelings of deprivation but may also prompt them to resort to relational aggression as a means of retaliation. Finally, the propensity for relational aggression among narcissists is closely tied to feelings of relative deprivation. This finding aligns with previous research suggesting that, despite variations in specific traits, narcissism as a complex personality trait shares similarities in terms of power, self-interest, and noncompliance (Wink, 1991; Miller and Campbell, 2008; Miller et al., 2017). Both grandiose and vulnerable narcissists are particularly concerned with their social status and interpersonal dynamics. Therefore, when they encounter difficulties or external threats, they experience a heightened sense of deprivation and may turn to relational aggression as a means to preserve or elevate their desired status (Pincus and Lukowitsky, 2010).

From a cognitive research perspective, this study reveals that hostile attribution bias significantly mediates the relationship between Machiavellianism, psychopathy, and relational aggression, supporting hypothesis H3. The findings suggest that individuals high in Machiavellianism, psychopathy, and narcissism are more likely to have hostile attribution bias and to engage in relational aggression. First, Machiavellians’ negative views of human nature and the world may lead to hostile attribution bias, which is often expressed through relational aggression. Although previous related research has not sufficiently confirmed the association between Machiavellianism and hostile attribution bias, in this study, we found a connection between the two and further revealed the mediating effect of hostile attribution bias between Machiavellianism and relational aggression, providing new useful information for future research. Secondly, the relational aggression of psychopaths may also be related to hostile attribution bias. Specifically, due to the unique emotional deficits of psychopaths, this group often struggles to recognize non-dominant social cues, making it easy for them to interpret ambiguous social cues as hostility from others and respond with aggressive behavior. However, this pattern not only traps them in a more hostile environment but also forms or reinforces their hostile attribution bias in a vicious cycle, exacerbating the occurrence of aggressive behavior (Wallace et al., 1999). However, note that whereas narcissism correlates with hostile attribution bias, this bias does not significantly mediate the relationship between narcissism and relational aggression, aligning with previous findings (Law and Falkenbach, 2018). Some scholars have suggested that whereas there is a consistent association between narcissism and hostile attribution bias, this association may only exist in vulnerable narcissism, rather than in grandiose narcissism (Hansen-Brown and Freis, 2021). Specifically, vulnerable narcissists, due to their heightened sensitivity to the behaviors and words of others, may perceive the external world or others as untrustworthy and tend to view social relationships through the lens of hostile attribution bias; whereas grandiose narcissists often display more confidence in social situations than vulnerable narcissists and tend to see others as loyal audiences or as tools for social gain.

In addition, this study, building on previous research, found that relative deprivation positively predicts hostile attribution bias, supporting research hypothesis H4. The results suggest that the more individuals perceive relative deprivation, the higher the level of hostile attribution bias. According to the integrated cognitive model, when individuals subjectively perceive threat signals, it activates their hostile cognitive schema. This activation prompts them to attribute hostility to social cues, which can lead to aggressive behavior. As aggression frequency increases, their hostile cognitive expectations are reinforced, resulting in defects in recognizing social cues (i.e., others’ language, expressions, and actions), which triggers hostile attribution bias (Crick and Dodge, 1994; Dodge et al., 2015). This indicates that for individuals with a history of victimization, adverse social situations not only lead to a perception of relative deprivation but also reactivate their pre-existing hostile cognitive schema, contributing to hostile attribution bias. Prolonged exposure to adverse social situations may also reinforce the perception of relative deprivation and potentially lead to the cognitive defect of hostile attribution bias. Moreover, it is notable that relative deprivation and hostile attribution bias act as serial mediators between the Dark Triad traits—Machiavellianism, psychopathy, narcissism, and relational aggression. This implies that individuals with these traits may experience a heightened sense of relative deprivation and increased hostile attribution bias when faced with unfair treatment or unfavorable social circumstances. In response, they may engage in relational aggression as a means to alter their negative situation.

### Theoretical implications

This study makes several theoretical contributions to the existing field. It is the first in-depth investigation of the relationship between the Dark Triad and relational aggression using a large sample. This not only verifies the connection between the two but also highlights the significant role of subjective perception (situational factors) and cognition (internal state). First, we propose that relative deprivation arises not only from material needs such as economic level, survival resources, and social conditions (Moscatelli et al., 2014; Wickham et al., 2014; Nieuwenhuis et al., 2017; Kassab et al., 2021) but also from social psychological needs, including self-belonging, value recognition, and emotional needs. Therefore, this study considers relative deprivation as a mediating factor, broadening the research perspective on the Dark Triad and relational aggression and offering new insights for related fields. Second, although previous studies have consistently verified the correlation between hostile attribution bias and the Dark Triad, as well as relational aggression, research on the role of hostile attribution bias in this relationship is limited. Thus, this study addresses this gap by treating hostile attribution bias as a mediating factor, deepening the understanding of the interplay among the three and providing valuable information for subsequent research. Finally, building on prior research, this study confirms the predictive effect of relative deprivation on hostile attribution bias and uncovers their serial mediating role in the relationship between the Dark Triad and relational aggression, offering new references for future research in this area.

### Practical implications

In addition, this study holds practical significance. Firstly, it underscores the critical role of the Dark Triad in individual social relationships and enhances our understanding of malignant interpersonal relationships from the perspective of subjective perception and cognition. Secondly, this research offers new reference information for intervention measures and strategies aimed at addressing malignant interpersonal relationships. This can assist mental health professionals or social workers in developing targeted intervention plans to mitigate harmful social interactions. For example, in clinical practice, therapeutic techniques focused on slowing down information processing have proven effective in curbing aggressive impulses in patients with hostile attribution bias (Hudley and Graham, 1993). Concurrently, cognitive behavioral therapy is a viable option, as it aids individuals in identifying and overcoming negative thinking and dysfunctional beliefs, thereby guiding them toward more constructive problem-solving strategies instead of aggression (Sukhodolsky et al., 2005).

### The limitations and future research directions

It should be noted that this study has some limitations. Firstly, the SD3 was used to measure Machiavellianism, psychopathy, and narcissism, which may not fully capture the common and unique factors of the three personality traits and their relationship to relational aggression. Therefore, future research may benefit from using other tools, such as the PID-5, to gain a better understanding of the connection between the Dark Triad and relational aggression. Secondly, this study did not thoroughly explore the functionality of relational aggression, such as differentiating between proactive and reactive forms. Future research into the functionality of the Dark Triad and relational aggression may provide deeper insights into their relationship. Thirdly, although the sample size of this study is relatively large, the data were collected within the specific cultural context of China. Given China’s collectivist tendencies and the importance placed on social relationships, the findings may vary compared to other regions. Therefore, future research should consider collecting data from a variety of countries and regions to validate the conclusions. Finally, and most importantly, the current findings are based on cross-sectional data, which does not allow for the accurate inference of causal relationships between variables. Longitudinal data may be necessary to verify the current findings in future research.

## Conclusion

Machiavellianism, psychopathy, and narcissism significantly predict relational aggression. The mediating role of relative deprivation is significant in the relationship between these personality traits and relational aggression. Hostile attribution bias plays a significant mediating role between Machiavellianism, psychopathy, and relational aggression, but not between narcissism and relational aggression. Furthermore, the sequential mediating role of both relative deprivation and hostile attribution bias is significant in the relationship between the Dark Triad traits (Machiavellianism, psychopathy, narcissism) and relational aggression.

## Data Availability

The raw data supporting the conclusions of this article will be made available by the authors, without undue reservation.
